# Impact of neighborhood resources on cardiovascular disease: a nationwide six-year follow-up

**DOI:** 10.1186/s12889-016-3293-5

**Published:** 2016-07-26

**Authors:** Susanna Calling, Xinjun Li, Naomi Kawakami, Tsuyoshi Hamano, Kristina Sundquist

**Affiliations:** 1 Department of Clinical Sciences, Center for Primary Health Care Research, Skåne University Hospital, Lund University, Clinical Research Centre (CRC), Building 28, floor 11, Jan Waldenströms gata 35, 205 02, Malmö, Sweden; 2 Waseda Institute of Sport Sciences, Waseda University, Tokorozawa Saitama, Japan; 3 Center for Community-based Health Research and Education (COHRE), Organization for the Promotion of Project Research, Shimane University, Izumo, Japan; 4 Stanford Prevention Research Center, Stanford University, Stanford, CA USA

**Keywords:** Cardiovascular disease, Follow-up study, Multilevel analysis, Neighborhood

## Abstract

**Background:**

Living in a socially deprived neighborhood is associated with lifestyle risk factors, e.g., smoking, physical inactivity and unhealthy diet, as well as an increased risk of cardiovascular disease, i.e., coronary heart disease and stroke. The aim was to study whether the odds of cardiovascular disease vary with the neighbourhood availability of potentially health-damaging and health-promoting resources.

**Methods:**

A nationwide sample of 2 040 826 men and 2 153 426 women aged 35–80 years were followed for six years for first hospitalization of coronary heart disease or stroke. Neighborhood availability of health-damaging resources (i.e., fast-food restaurants and bars/pubs) and health-promoting resources (i.e., health care facilities and physical activity facilities) were determined by use of geographic information systems (GIS).

**Results:**

We found small or modestly increased odds ratios (ORs) for both coronary heart disease and stroke, related to the availability of both health-damaging and health-promoting resources. For example, in women, the unadjusted OR (95 % confidence interval) for stroke in relation to availability of fast-food restaurants was 1.18 (1.15–1.21). Similar patterns were observed in men, with an OR = 1.08 (1.05–1.10). However, the associations became weaker or disappeared after adjustment for neighborhood-level deprivation and individual-level age and income.

**Conclusions:**

This six year follow-up study shows that neighborhood availability of potentially health-damaging as well as health-promoting resources may make a small contribution to the risk of coronary heart disease and stroke. However, most of these associations were attenuated or disappeared after adjustment for neighborhood-level deprivation and individual-level age and income. Future studies are needed to further examine factors in the causal pathway between neighborhood deprivation and cardiovascular disease.

**Electronic supplementary material:**

The online version of this article (doi:10.1186/s12889-016-3293-5) contains supplementary material, which is available to authorized users.

## Background

Cardiovascular disease (CVD) is still the leading cause of death in Sweden and many other Western countries, even if there has been a marked decrease in the incidence rates during the last decades [1]. The reasons behind CVD are multifactorial, including metabolic, sociodemographic and lifestyle factors, e.g., smoking, unhealthy diet and physical inactivity [2, 3]. The prevalence of obesity has markedly increased during the last decades [4]. Efforts have been made to counteract the unfavorable trend by interventions to increase physical activity and reduce intake of unhealthy food in the population [5–7]. Several studies have shown associations between modifiable risk factors, e.g., physical inactivity and obesity, and living in a socially deprived neighborhood [8, 9].

Furthermore, neighborhood-level socioeconomic deprivation is associated with CVD, and the reasons behind this association are not fully explored [10–12]. Lifestyle risk factors could lie in the pathway, but the underlying mechanisms are poorly understood. Studies linking neighborhood environment and CVD risk factors have yielded mixed results and have most often been limited by cross-sectional designs and same-source bias [6, 7, 13, 14]. It has been debated whether deprived neighborhoods have higher density of health-damaging resources, such as fast food restaurants, and lower access to health-promoting resources, such as health care services and physical activity facilities [15]. Previous studies have found that the density of fast-food restaurants is associated with neighborhood-level deprivation [16, 17]. However, no clear associations have been found between lack of health-promoting resources and neighborhood-level deprivation [18, 19]. Moreover, several cross-sectional studies have found that individuals living in areas with high density of fast-food restaurants were more likely to be obese, but some studies could not confirm this association [20–22]. Contradictory results have been reported for a potential association between density of physical activity facilities and level of physical activity [23, 24].

Studies trying to disentangle a possible association between neighborhood resources and CVD as endpoint have found no clear associations [25–28]. Moreover, most studies did not have a longitudinal multilevel approach. In 2011, Kawakami et al. performed a multilevel analysis of a large Swedish study sample during two years of follow-up, which showed weak associations between coronary heart disease (CHD) and neighborhood availability of potentially health-damaging and health-promoting goods, services and resources, but the associations disappeared after controlling for neighborhood-level deprivation and individual-level age and income [29]. In a similar Swedish study in 2013, Hamano et al. showed weak associations between stroke and neighborhood resources, which partly remained after adjustments for individual-level sociodemographic factors and neighborhood-level deprivation [30]. In these two studies, it was concluded that a longer follow-up was needed to further disentangle the possible association between neighborhood resources and CVD. The aim of the present study was to clarify whether neighborhood resources make any contribution to cardiovascular risk during a six-year follow-up and it represents the largest and longest follow up so far using the “hard” outcomes coronary heart disease and stroke.

The novelty of the present study is the availability of a nationwide sample of more than 4 million individuals with an extended follow-up period of six years, and a longitudinal multilevel approach. The first aim was to examine, in men and women separately, whether the risk of CHD and stroke was associated with neighborhood availability of potentially health-damaging resources, such as fast-food restaurants and bars/pubs, as well as health-promoting resources, such as physical activity facilities and health care services. The second aim was to study whether controlling for neighborhood-level deprivation and individual-level age and income affected the results.

## Methods

### Study population

The study population included a nationwide sample of 2 040 826 men and 2 153 426 women aged 35–80 years. The same study population was used in our previous paper [30] but the present study was based on an extended follow-up of six years. Data were retrieved from national Swedish registers, containing information on nationwide individual-level medical diagnoses from the Swedish Hospital Discharge Register (obtained from the National Board of Health and Welfare) and the Cause of Death Register. These data were linked to registers obtained from Statistics Sweden, the Swedish Government-owned statistics bureau, which provided individual-level data on socioeconomic factors, such as age and income.

### Outcome variable

All individuals were followed between 1 January 2005 and 31 December 2010 for their first hospitalization of CHD or stroke as well as mortality for CHD or stroke. Men and women with either pre-existing CHD or stroke, defined as hospitalization ≤ 5 years before the start of the study, were excluded. In an additional analysis, we excluded those individuals with pre-existing CHD or stroke ten years prior start of the study. The disease codes were based on the 10^th^ version of the International Classification of Diseases (ICD-10) and included the following diagnoses:

- CHD
  I20, angina pectoris; I21, acute myocardial infarction; I22, subsequent myocardial infarction; I23, complications owing to acute cardiac infarction; I24, other acute forms of CHD; and I25, chronic CHD.
- Stroke
  I60, subarachnoidal haemorrhage; I61-62, hemorrhagic stroke; I63 cerebral infarction; I64, acute cerebrovascular disease not specified as hemorrhagic or infarction; I65-I65, occlusion and stenosis of precerebral and cerebral arteries; I67-I68, other cerebrovascular diseases; and I69, late effects of cerebrovascular disease.

### Predictor variable

Four categories of neighborhood goods, services and resources were selected to represent potentially health-damaging and health-promoting resources [31], i.e.,:

1. fast-food restaurants (e.g., pizzerias and hamburger outlets)
2. bars/pubs
3. health care facilities (e.g., health care centers, public hospitals, dentists, and pharmacies)
4. physical activity facilities (e.g., swimming pools, gyms, and ski facilities).

The ready-to-use nationwide GIS (Geographic Information Systems) dataset of business contacts (i.e., goods, services and resources) was provided to us by the Swedish company *Teleadress*, which is a leading provider of Swedish contact information [32]. *Teleadress* provides information on practically all businesses and services in Sweden with a registered telephone number and/or businesses that have provided information about their existence to the company. Both government and private entities are included. The database is updated continuously and has a high level of completeness of the data [31]. Data was drawn from 2005.

Neighborhood availability of the four categories was measured in 2005 as counts per predefined area called SAMS (Small Area Market Statistics, provided by Statistics Sweden, see below) by the use of GIS. Availability was defined as the presence within the SAMS unit of at least one feature for the category in question. Presence yes/no was chosen to define neighborhood availability rather than linear density because the large majority had no access to the studied resources [31]. The correlation between the presence of the different types of resources was low and varied between 0.2 and 0.4.

Neighborhood of residence was provided to us by Statistics Sweden via data from the National Land Survey. The home addresses of all individuals had been previously geocoded, allowing us to identify the SAMS units in which the participants lived. However, the researchers in the present study had no access to the home addresses of the individuals, in order to ensure all individuals’ integrity. SAMS units were used as proxies for neighborhoods and has been used previously [11, 33]. Each unit contains an average of around 1000 residents in all Sweden and around 2000 in Stockholm. This study examined only those SAMS units that overlap with ‘localities’ or urban areas. In Sweden, ‘localities’ (which are defined by Statistics Sweden every fifth year) represent any village, town or city with a minimum of 200 residents and adjacent areas where the houses are no more than 200 m apart [34]. We chose to include only SAMS units overlapping with localities because more rural SAMS units have very few goods, services and resources. In 2005, 1940 Swedish localities were identified by Statistics Sweden. GIS were used to overlay the SAMS boundaries with the locality boundaries. Of a total of 9 617 SAMS units in Sweden, 7 945 overlapped with localities and were therefore selected. SAMS units with fewer than 50 people were excluded on the basis that they might yield unreliable statistical estimates in the calculation of the neighborhood deprivation index. A final total of 7 033 SAMS units was included in the present study.

In order to investigate the individuals’ immediate neighborhood, we also used buffer zones. Statistics Sweden provided information about the individuals’ land coordinates in squares of 100 × 100 m, ensuring the integrity of the individuals. For each individual, a buffer zone with a radius of 1000 m was applied, i.e., a distance that most people are willing to walk. Most previous studies use ½ mile (~800 m) and/or 1 mile (~1600 m), which corresponds relatively well to the present study [35]. The number of goods, services and resources within the buffer zones was calculated using GIS. Availability was defined as the presence within the buffer zone of at least one feature for the category in question.

### Covariates

All covariates were measured at baseline in 2005.

*Individual-level age* was categorized as 35–44, 45–54, 55–64, 65–74 and 75–80 years.

*Individual-level family income* was calculated from the annual family income divided by the number of family members and took into account the ages of the people in the family. The variable was categorized as empirical quartiles of the distribution.

*Neighborhood-level deprivation* was included in the analysis as a covariate, as previous research has shown that it is associated with an increased risk of CHD [10–12]. This is in line with previous studies [29, 30]. A summary measure was used to characterize neighborhood-level deprivation. We identified deprivation indicators used by past studies to characterize neighborhood environments and then used a principal components analysis to select deprivation indicators in the national registers. The following four variables were selected for those aged 25–64: low educational status (<10 years of formal education); low income (income from all sources, including that from interest and dividends, defined as less than 50 % of individual median income); unemployment (not employed, excluding full-time students, those completing compulsory military service, and early retirees); and social welfare recipient. Each of the four variables loaded on the first principal component with similar loadings (+.47 to + .53) and explained 52 % of the variation between these variables. A z score was calculated for each SAMS unit, and these scores were summed to create the index. The z scores, weighted by the coefficients for the eigenvectors, were then summed to create the index. The index was categorized into three groups: below one standard deviation (SD) from the mean (low deprivation), above one SD from the mean (high deprivation), and within one SD of the mean (moderate deprivation). Higher scores reflect more deprived neighborhoods. The rationale for this categorisation was that it is easier to interpret results in categories and accordingly identify populations for intervention. The neighborhood deprivation index was based on those individuals living in the neighborhood aged 25–64 years as individuals in those ages are supposed to be more socioeconomically active and thus have a stronger impact on the neighborhood than, for example, young persons and retirees. The variables used in the index are most relevant among those in working ages, which is around 25–64 years of age in Sweden. All individuals in the study population (ages 35–80 years) were assigned a neighborhood score.

### Statistics

Age-standardized incidence proportions (proportions of subjects who became cases among those who entered the study time interval) were calculated separately for men and women by direct age standardization using 10-year age groups, with the entire Swedish population of men or women aged 35–80 as the standard population.

Multilevel (hierarchical) logistic regression models were created with incidence proportions as the outcome variables. The analyses were performed using MLwiN. Multilevel logistic regression models were used in the computing process to help our multilevel models to converge, as the large dataset did not converge when we used multilevel Cox proportional hazards models. These models are a good approximation of multilevel Cox proportional hazards models under certain conditions, such as ours, i.e., a large sample size, low incidence rates, risk ratios of moderate size and relatively short follow-up [36]. Previous studies have used a similar approach [8]. For comparison, we made a sensitivity analysis by creating ordinary Cox proportional hazards models (Additional file 1: Tables S1 and S2). These models gave similar results. First, we created models that only included neighborhood availability of each of the four categories of neighborhood goods, services and resources in order to determine the crude odds ratios (OR) for CHD and stroke with 95 % confidence intervals (CI). Next, we created a model that included neighborhood-level deprivation. The third and final model included neighborhood availability, neighborhood-level deprivation and individual-level age and income.

Random intercept multilevel logistic regression models were used to allow for the clustering of individuals within neighborhoods and to estimate the variance in CVD risk that is attributable to neighborhood characteristics. This approach was used to estimate the intraclass correlation coefficient (ICC), the proportion of variance in the outcome attributable to differences between individuals in different neighborhoods in contrast to differences between individuals within the same neighborhood [37]. The ICC was estimated by applying the latent variable method as exemplified by:

$$ ICC=\frac{Vn}{Vn+{\pi}^2/3} $$

where Vn is the variance between neighborhoods and π^2^/3 is the variance between individuals.

The proportion of the second level variance explained by the different variables was calculated as:

$$ {V}_{EXPLAINED}=\frac{V_0-{V}_1}{V_0}\times 100, $$

where Vo is the age adjusted variance in the initial model and V_1_ is the second level variance in the different models.

Cross-level interaction tests were performed. The interaction tests were performed to examine potential interactions between neighborhood deprivation and availability of the four categories of resources. The results showed no meaningful interactions (data not shown).

## Results

Table 1 shows the distribution of the study population and the number of CHD and stroke events in relation to neighborhood deprivation and availability of the four types of resources, in men and women separately. In men, 12.7 % had access to bars/pubs in their neighborhood, 45.4 % to fast food restaurants, 38.5 % to healthcare services and 41.0 % to physical activity facilities. In women, similar proportions were found. Incidence of CVD increased with higher level of neighborhood deprivation in all subgroups; however, there were no large differences in relation to availability of the four resources. In men, the OR (95 % CI) in relation to high neighborhood deprivation compared to low deprivation was 1.39 (1.35–1.42) for CHD, and 1.28 (1.24–1.32) for stroke, adjusted for individual-level age and income. The corresponding ORs in women were 1.60 (1.55–1.66) for CHD and 1.36 (1.31–1.41) for stroke (data not shown in tables).

**Table 1:** Distribution of population and age-standardized incidence proportions of cardiovascular disease, by neighborhood-level deprivation

	Population	Distribution	Number of CHD events	Neighborhood deprivation			Number of stroke events	Neighborhood deprivation		
	No.	(%)		Low (*n* = 467965)	Moderate (*n* = 1220698)	High (*n* = 352163)		Low (*n* = 467965)	Moderate (*n* = 1220698)	High (*n* = 352163)
Men										
Total population	2 040 826		100 147	3.9	5.0	5.8	51 802	2.0	2.6	3.0
Bars/pubs										
No availability	1 782 278	87.3	86 877	3.9	5.0	5.8	44 762	2.0	2.6	3.0
Availability	258 548	12.7	13 270	3.6	5.1	5.6	7040	2.2	2.7	2.9
Fast food restaurants										
No availability	1 113 878	54.6	53 589	3.9	5.0	5.9	27 333	2.0	2.6	2.9
Availability	926 948	45.4	46 558	3.9	5.1	5.7	24 469	2.0	2.7	3.0
Health care facilities										
No availability	1 254 981	61.5	59 663	3.9	5.0	5.8	30 469	2.0	2.6	3.0
Availability	785 845	38.5	40 484	3.7	5.1	5.8	21 333	2.0	2.7	3.0
Physical activity facilities										
No availability	1 204 920	59.0	58 110	3.8	5.0	5.9	30 037	2.0	2.6	2.9
Availability	835 906	41.0	42 037	4.0	5.1	5.7	21 765	2.1	2.6	3.0
Women										
Total population	2 153 426		54 441	1.7	2.6	3.4	42 113	1.4	2.0	2.4
Bars/pubs										
No availability	1 863 276	86.5	46 218	1.7	2.6	3.4	35 618	1.4	2.0	2.4
Availability	290 150	13.5	8223	1.6	2.6	3.2	6495	1.4	2.1	2.4
Fast food restaurants										
No availability	1 147 263	53.3	26 945	1.7	2.6	3.4	20 747	1.4	2.0	2.4
Availability	1 006 163	46.7	27 496	1.7	2.6	3.4	21 366	1.4	2.1	2.5
Health care facilities										
No availability	1 290 150	59.9	30 066	1.7	2.6	3.4	23 372	1.4	2.0	2.4
Availability	863 276	40.1	24 375	1.6	2.6	3.4	18 741	1.4	2.0	2.4
Physical activity facilities										
No availability	1 266 217	58.8	31 196	1.7	2.6	3.4	23 960	1.4	2.0	2.4
Availability	887 209	41.2	23 245	1.7	2.6	3.4	18 153	1.5	2.0	2.5

Distribution of population, number of coronary heart disease (CHD) and stroke events, and age-standardized incidence (%) by neighborhood-level deprivation

Table 2 shows the associations between incidence of CHD/stroke and neighborhood availability of the four resources in the SAMS units. The ORs with 95 % CIs are presented for CHD and stroke, in relation to neighborhood availability. In the unadjusted models (Model 1), availability of all resources, both health-promoting and health-damaging, showed statistically significant increased ORs for both CHD and stroke. The associations were stronger in women than in men. The highest ORs were found in women for fast-food restaurants (OR for CHD = 1.17 [95 % CI: 1.13–1.20], ICC 0.043) and for health care facilities (OR for CHD = 1.22 [95 % CI: 1.19–1.26], ICC 0.042). After adjustments for neighborhood-level deprivation (Model 2) and individual-level age and income (Model 3), the associations became weaker or disappeared. The associations for stroke were generally somewhat stronger than for CHD. For CHD, the increased ORs remained only for neighborhood availability of fast-food restaurants and health care facilities in women, after full adjustments.

**Table 2:** Associations between cardiovascular disease and neighborhood availability of the four resources, small area market statistics

	Men (*N* = 2 040 826)									Women (*N* = 2 153 426)								
	Model-1			Model-2			Model-3			Model-1			Model-2			Model-3		
	OR	95 % CI		OR	95 % CI		OR	95 % CI		OR	95 % CI		OR	95 % CI		OR	95 % CI	
Coronary heart disease																		
Bars/pubs																		
Availability	**1.04**	**1.01**	**1.08**	1.01	0.98	1.04	1.00	0.97	1.02	**1.13**	**1.08**	**1.17**	**1.08**	**1.04**	**1.13**	0.99	0.96	1.02
No availability	1			1			1			1			1			1		
Fast-food restaurants																		
Availability	**1.05**	**1.03**	**1.07**	**1.01**	**1.00**	**1.03**	1.01	0.99	1.02	**1.17**	**1.13**	**1.20**	**1.10**	**1.08**	**1.13**	**1.02**	**1.00**	**1.04**
No availability	1			1			1			1			1			1		
Healthcare facilities																		
Availability	**1.10**	**1.07**	**1.12**	**1.05**	**1.03**	**1.07**	1.01	0.99	1.02	**1.22**	**1.19**	**1.26**	**1.15**	**1.12**	**1.18**	**1.03**	**1.00**	**1.05**
No availability	1			1			1			1			1			1		
Physical activity facilities																		
Availability	**1.04**	**1.02**	**1.06**	**1.03**	**1.01**	**1.05**	1.01	0.99	1.03	**1.05**	**1.02**	**1.08**	**1.04**	**1.02**	**1.07**	1.01	0.99	1.03
No availability	1			1			1			1			1			1		
Stroke																		
Bars/pubs																		
Availability	**1.08**	**1.04**	**1.12**	**1.05**	**1.01**	**1.08**	**1.03**	**1.00**	**1.06**	**1.17**	**1.12**	**1.22**	**1.21**	**1.16**	**1.26**	1.02	0.99	1.05
No availability	1			1			1			1			1			1		
Fast-food restaurants																		
Availability	**1.08**	**1.05**	**1.10**	**1.04**	**1.02**	**1.06**	**1.03**	**1.01**	**1.05**	**1.18**	**1.15**	**1.21**	**1.13**	**1.10**	**1.16**	**1.03**	**1.01**	**1.06**
No availability	1			1			1			1			1			1		
Healthcare facilities																		
Availability	**1.12**	**1.10**	**1.15**	**1.08**	**1.06**	**1.10**	**1.03**	**1.01**	**1.05**	**1.21**	**1.17**	**1.24**	**1.15**	**1.12**	**1.18**	1.02	0.99	1.04
No availability	1			1			1			1			1			1		
Physical activity facilities																		
Availability	**1.05**	**1.02**	**1.07**	**1.03**	**1.01**	**1.06**	1.01	0.99	1.03	**1.08**	**1.05**	**1.11**	**1.06**	**1.04**	**1.09**	**1.03**	**1.01**	**1.05**
No availability	1			1			1			1			1			1		

Models showing associations between coronary heart disease and stroke and neighborhood availability of potentially health-damaging and health-promoting goods, services and resources in the “small area market statistics”, Boldface = statistically significant (*p*>0.005)

ICC was generally low and varied between 0.004 and 0.010 in the fully adjusted models, indicating that the majority of variance in CVD risk was attributed to within-neighborhood rather than between-neighborhood differences.

Table 3 shows the associations between incidence of CHD/stroke and the “immediate” neighborhood availability of the four resources in the buffer zones (radius 1000 m). The associations were similar to the results in the SAMS units, but the ORs in the buffer zones were somewhat stronger. For stroke, there were after full adjustments (Model 3) statistically significant increased ORs in both men and women, (e.g., OR in men = 1.06 [95 % CI: 1.04–1.08]) for fast-food restaurants and OR = 1.07 [95 % CI: 1.05–1.09] for health care facilities), with the exception of OR for stroke in relation to physical activity resources in women, where no association was found.

**Table 3:** Associations between cardiovascular disease and “immediate” neighborhood availability of the four resources, buffer zones

	Men (*N* = 2 040 826)									Women (*N* = 2 153 426)								
	Model-1			Model-2			Model-3			Model-1			Model-2			Model-3		
	OR	95 % CI		OR	95 % CI		OR	95 % CI		OR	95 % CI		OR	95 % CI		OR	95 % CI	
Coronary heart disease																		
Bars/pubs																		
Availability	**1.03**	**1.01**	**1.05**	1.01	0.99	1.02	0.99	0.97	1.00	**1.18**	**1.15**	**1.21**	**1.14**	**1.11**	**1.16**	0.99	0.97	1.02
No availability	1			1			1			1			1			1		
Fast-food restaurants																		
Availability	**1.06**	**1.04**	**1.08**	**1.04**	**1.02**	**1.05**	1.00	0.98	1.01	**1.24**	**1.21**	**1.27**	**1.21**	**1.18**	**1.23**	**1.02**	**1.00**	**1.04**
No availability	1			1			1			1			1			1		
Healthcare facilities																		
Availability	**1.07**	**1.06**	**1.09**	**1.04**	**1.03**	**1.06**	1.00	0.86	1.17	**1.27**	**1.24**	**1.30**	**1.22**	**1.19**	**1.25**	**1.02**	**1.00**	**1.04**
No availability	1			1			1			1			1			1		
Physical activity facilities																		
Availability	**1.03**	**1.01**	**1.05**	**1.01**	**1.00**	**1.03**	0.99	0.97	1.01	**1.14**	**1.11**	**1.16**	**1.11**	**1.08**	**1.13**	0.98	0.96	1.00
No availability	1			1			1			1			1			1		
Stroke																		
Bars/pubs																		
Availability	**1.10**	**1.07**	**1.12**	**1.07**	**1.05**	**1.10**	**1.05**	**1.03**	**1.08**	**1.21**	**1.18**	**1.25**	**1.18**	**1.15**	**1.21**	**1.03**	**1.00**	**1.05**
No availability	1			1			1			1			1			1		
Fast-food restaurants																		
Availability	**1.11**	**1.09**	**1.14**	**1.10**	**1.07**	**1.12**	**1.06**	**1.04**	**1.08**	**1.27**	**1.24**	**1.31**	**1.25**	**1.21**	**1.28**	**1.04**	**1.02**	**1.07**
No availability	1			1			1			1			1			1		
Healthcare facilities																		
Availability	**1.15**	**1.12**	**1.17**	**1.12**	**1.10**	**1.15**	**1.07**	**1.05**	**1.09**	**1.27**	**1.24**	**1.30**	**1.23**	**1.21**	**1.26**	**1.02**	**1.00**	**1.05**
No availability	1			1			1			1			1			1		
Physical activity facilities																		
Availability	**1.09**	**1.07**	**1.11**	**1.07**	**1.05**	**1.09**	**1.05**	**1.03**	**1.07**	**1.18**	**1.15**	**1.21**	**1.16**	**1.13**	**1.19**	1.02	0.99	1.04
No availability	1			1			1			1			1			1		

Models showing associations between coronary heart disease and stroke and neighborhood availability of potentially health-damaging and health-promoting goods, services and resources in the “buffer zones”, Boldface = statistically significant (*p*>0.005)

We also made an additional analysis with only mortality from CHD and stroke as an outcome. The results were overall similar to the main results; however, no associations were found for stroke mortality in women.

In a separate analysis, we excluded individuals with hospitalization of CVD ten years prior to the start of the follow-up; however, the results from this sensitivity analysis were very similar to the main results in the present study.

## Discussion

The main finding of the present study with six years’ follow-up is that neighborhood availability of potentially health-damaging as well as health-promoting resources contributes to small but increased odds of CHD and stroke. However, the major part of the increased odds was related to neighborhood-level deprivation and individual-level age and income, which is in line with the results of our previous studies with shorter follow-up [29, 30]. The results indicate that vicinity of health-damaging resources, i.e., fast food and bars/pubs, may make a small contribution to an increased cardiovascular risk, probably through an unhealthier lifestyle. However, the effect seems to be small and this research area is not fully understood. Earlier studies have shown that the built environment can affect obesity and physical activity, but the relationship is complex and operates through many mediating variables, such as socio-economic characteristics, personal and cultural variables, and safety in the built environment [6, 7]. Neighborhood availability of fast food restaurants has been shown to be related to increased fast food consumption [38], and density of fast-food restaurants is associated with CVD [25, 27, 28]. However, we do not know whether the studied individuals choose resources that are not in their immediate neighborhood. As the studied health-damaging resources were only weak predictors of CVD, other neighborhood mechanisms may more strongly influence the increased risk of CVD in socioeconomically deprived neighborhoods, e.g., social disintegration (criminality and unemployment) [39], low social capital [40] and stress [41].

Concerning potentially health-promoting resources, the present study showed that high neighborhood availability, especially of health care facilities, was also associated with increased risk of CVD. This supports the conclusion that the increased cardiovascular risk in deprived neighborhoods is mainly associated with individual socioeconomic characteristics as well as the neighborhood-level deprivation itself rather than the availability of neighborhood resources, and that it is unlikely that neighborhood resources have a large influence on cardiovascular risk. Generally, the associations between neighborhood availability of the studied resources and CVD were somewhat stronger for stroke than for CHD. Previous research indicates that genetic influence on stroke risk is weaker than for other cardiovascular manifestations [42, 43], suggesting that lifestyle and other modifiable risk factors may have stronger influence on the disease development. However, when studying stroke mortality separately we found no associations in women. As mortality is an outcome that comes later than lifestyle factors and cardiovascular disease, it is also more difficult to interpret. For example, for mortality, genetic factors may play an important role.

### Strengths and limitations

Our study had several strengths. We were able to follow a large study sample of the entire Swedish population for CVD, which so far only has been possible in our nationwide study population. In the present study, we were able to extend the follow-up period to six years to better follow the cardiovascular risk than in our previous studies [29, 30]. Six years of follow-up is still short enough to ensure that most people stayed in their neighborhood, so that any potential changes in availability of resources were minor. Preliminary unpublished data from Sweden shows that only 1 % of middle-aged people change neighborhood socioeconomic status during a 10-year period.

The multi-level approach and adjustments for neighborhood-level deprivation and individual-level covariates measured at baseline are also strengths of the present study. Moreover, we had access to valid geographic information about neighborhood availability of several different resources. The data on neighborhood resources are very complete, as Teleadress provides information on practically all businesses in Sweden [32]. Finally, our use of small neighborhood units (SAMS and individuals’ buffer zones) increases the probability that the residents were actually exposed to the studied resources.

However, our study also had certain limitations. We do not know whether the residents actually utilized the studied resources in their neighborhoods, or whether the individuals changed neighborhoods during the follow-up period. Another limitation was that the neighborhoods were based on geographic areas and may therefore not have corresponded perfectly with the individuals’ social environment. Moreover, it is likely that residual confounding exists. We had no data on lifestyle factors, e.g., unhealthy diet, smoking and physical inactivity, which are important and modifiable cardiovascular risk factors.

## Conclusions

In this large-scale study with six years follow up, we found increased but small odds of CHD and stroke associated with neighborhood availability of potentially health damaging fast-food restaurants and bars/pubs, as well as availability of potentially health-promoting healthcare services and physical activity facilities. Although planning of residential areas should aim to promote neighborhood resources that encourage a healthy lifestyle, the present results suggest that neighborhood resources are unlikely to make an important contribution to variations in cardiovascular disease by neighborhood deprivation. More studies in this area are needed to further examine potential causal factors in the pathway between neighborhood deprivation and CVD.

## Abbreviations

CHD, coronary heart disease; CI, confidence interval; CVD, cardiovascular disease; GIS, geographic information systems; ICC, intraclass correlation coefficient; OR, odds ratio; SAMS, Small Area Market Statistics; SD, standard deviation

## Additional file

Additional file 1: Table S1. Models showing associations between coronary heart disease and stroke and neighborhood availability of potentially health-damaging and health-promoting goods, services and resources in the “small area market statistics”; Cox regression. **Table S2.** Models showing associations between coronary heart disease and stroke and neighborhood availability of potentially health-damaging and health-promoting goods, services and resources in the “buffer zones”; Cox regression. (DOCX 22 kb)
