# Supplementary material for: Impact of neighborhood resources on cardiovascular disease: a nationwide six-year follow-up
Source: BMC Public Health. 2016 Jul 26;16:634. doi: 10.1186/s12889-016-3293-5 (PMC4960746; doi:10.1186/s12889-016-3293-5)
Supplement: Additional file 1: Table S1. — Models showing associations between coronary heart disease and stroke and neighborhood availability of potentially health-damaging and health-promoting goods, services and resources in the “small area market statistics”; Cox regression. Table S2. Models showing associations between coronary heart disease and stroke and neighborhood availability of potentially health-damaging and health-promoting goods, services and resources in the “buffer zones”; Cox regression. (DOCX 22 kb) [file 12889_2016_3293_MOESM1_ESM.docx]

| **Supplementary Table 1. Association between cardiovascular disease and neighborhood resources in small area market statistics, Cox regression** | | | | | | | | | | | | | | | | | | | | | | | |  |  |
| --- | --- | --- | --- | --- | --- | --- | --- | --- | --- | --- | --- | --- | --- | --- | --- | --- | --- | --- | --- | --- | --- | --- | --- | --- | --- |
|  | Men (N=2 040 826) | | | | | | | | | | |  | Women (N=2 153 426) | | | | | | | | | | |  |  |
|  | Model-1 | | |  | Model-2 | | |  | Model-3 | | |  | Model-1 | | |  | Model-2 | | |  | Model-3 | | |  |  |
|  | HR | 95% CI | |  | HR | 95% CI | |  | HR | 95% CI | |  | HR | 95% CI | |  | HR | 95% CI | |  | HR | 95% CI | |  |  |
| **Coronary heart disease** |  |  |  |  |  |  |  |  |  |  |  |  |  |  |  |  |  |  |  |  |  |  |  |  |  |
| Bars/pubs | **1.06** | **1.04** | **1.08** |  | **1.02** | **1.00** | **1.04** |  | 1.01 | 0.99 | 1.03 |  | **1.15** | **1.12** | **1.17** |  | **1.09** | **1.07** | **1.12** |  | 1.00 | 0.98 | 1.02 |  |  |
| Fast-food restaurants | **1.05** | **1.04** | **1.06** |  | **1.01** | **1.00** | **1.02** |  | 1.01 | 0.99 | 1.02 |  | **1.17** | **1.15** | **1.19** |  | **1.10** | **1.08** | **1.12** |  | **1.02** | **1.00** | **1.04** |  |  |
| Healthcare facilities | **1.09** | **1.08** | **1.10** |  | **1.04** | **1.03** | **1.06** |  | **1.01** | **1.00** | **1.02** |  | **1.22** | **1.20** | **1.24** |  | **1.14** | **1.12** | **1.16** |  | **1.03** | **1.01** | **1.04** |  |  |
| Physical activity facilities | **1.04** | **1.03** | **1.06** |  | **1.03** | **1.02** | **1.05** |  | **1.01** | **1.00** | **1.03** |  | **1.07** | **1.05** | **1.08** |  | **1.05** | **1.03** | **1.07** |  | **1.02** | **1.00** | **1.03** |  |  |
|  |  |  |  |  |  |  |  |  |  |  |  |  |  |  |  |  |  |  |  |  |  |  |  |  |  |
| **Stroke** |  |  |  |  |  |  |  |  |  |  |  |  |  |  |  |  |  |  |  |  |  |  |  |  |  |
| Bars/pubs | **1.09** | **1.06** | **1.12** |  | **1.05** | **1.03** | **1.08** |  | **1.03** | **1.01** | **1.06** |  | **1.18** | **1.15** | **1.21** |  | **1.13** | **1.10** | **1.16** |  | 1.02 | 0.99 | 1.05 |  |  |
| Fast-food restaurants | **1.08** | **1.06** | **1.10** |  | **1.04** | **1.02** | **1.06** |  | **1.03** | **1.02** | **1.05** |  | **1.18** | **1.16** | **1.20** |  | **1.12** | **1.10** | **1.14** |  | **1.03** | **1.01** | **1.05** |  |  |
| Healthcare facilities | **1.12** | **1.10** | **1.14** |  | **1.08** | **1.06** | **1.10** |  | **1.04** | **1.02** | **1.05** |  | **1.20** | **1.18** | **1.23** |  | **1.14** | **1.12** | **1.16** |  | **1.02** | **1.00** | **1.04** |  |  |
| Physical activity facilities | **1.05** | **1.03** | **1.06** |  | **1.03** | **1.02** | **1.05** |  | **1.01** | **0.99** | **1.03** |  | **1.08** | **1.06** | **1.10** |  | **1.07** | **1.05** | **1.09** |  | **1.03** | **1.01** | **1.05** |  |  |
| OR: Odds ratio, CI: Confidence interval | | | | | | | | | | | | | | | | | | | | | | | |  |  |
| Neighborhoods are defined by predefined geographic units (small area market statistics). Model 1 is unadjusted. Model 2 is adjusted for neighborhood-level deprivation. Model 3 is adjusted for neighborhood-level deprivation and individual-level age and income. Bold type: | | | | | | | | | | | | | | | | | | | | | | | | | |
| Bold type: 95% CI does not include 1. | | |  |  |  |  |  |  |  |  |  |  |  |  |  |  |  |  |  |  |  |  |  |  |  |

| **Supplementary Table 2. Association between cardiovascular disease and neighborhood resources in buffer zones, Cox regression** | | | | | | | | | | | | | | | | | | | | | | | |  |  |
| --- | --- | --- | --- | --- | --- | --- | --- | --- | --- | --- | --- | --- | --- | --- | --- | --- | --- | --- | --- | --- | --- | --- | --- | --- | --- |
|  | Men (N=2040826) | | | | | | | | | | |  | Women (N=2153426) | | | | | | | | | | |  |  |
|  | Model-1 | | |  | Model-2 | | |  | Model-3 | | |  | Model-1 | | |  | Model-2 | | |  | Model-3 | | |  |  |
|  | HR | 95% CI | |  | HR | 95% CI | |  | HR | 95% CI | |  | HR | 95% CI | |  | HR | 95% CI | |  | HR | 95% CI | |  |  |
| **Coronary heart disease** |  |  |  |  |  |  |  |  |  |  |  |  |  |  |  |  |  |  |  |  |  |  |  |  |  |
| Bars/pubs | **1.03** | **1.01** | **1.04** |  | 1.00 | 0.98 | 1.01 |  | 0.99 | 0.98 | 1.00 |  | **1.18** | **1.16** | **1.20** |  | **1.12** | **1.10** | **1.14** |  | 1.00 | 0.98 | 1.01 |  |  |
| Fast-food restaurants | **1.04** | **1.02** | **1.05** |  | **1.01** | **1.00** | **1.03** |  | 1.00 | 0.99 | 1.01 |  | **1.24** | **1.22** | **1.27** |  | **1.19** | **1.16** | **1.21** |  | **1.02** | **1.00** | **1.04** |  |  |
| Healthcare facilities | **1.06** | **1.05** | **1.08** |  | **1.03** | **1.01** | **1.04** |  | 1.00 | 0.99 | 1.02 |  | **1.29** | **1.27** | **1.31** |  | **1.21** | **1.19** | **1.24** |  | **1.02** | **1.01** | **1.04** |  |  |
| Physical activity facilities | **1.02** | **1.01** | **1.04** |  | 1.00 | 0.98 | 1.01 |  | 0.99 | 0.98 | 1.01 |  | **1.16** | **1.14** | **1.18** |  | **1.11** | **1.09** | **1.13** |  | 0.98 | 0.97 | 1.00 |  |  |
|  |  |  |  |  |  |  |  |  |  |  |  |  |  |  |  |  |  |  |  |  |  |  |  |  |  |
| **Stroke** |  |  |  |  |  |  |  |  |  |  |  |  |  |  |  |  |  |  |  |  |  |  |  |  |  |
| Bars/pubs | **1.10** | **1.08** | **1.13** |  | **1.08** | **1.05** | **1.10** |  | **1.06** | **1.04** | **1.08** |  | **1.22** | **1.20** | **1.25** |  | **1.18** | **1.16** | **1.20** |  | **1.03** | **1.01** | **1.05** |  |  |
| Fast-food restaurants | **1.11** | **1.09** | **1.13** |  | **1.09** | **1.07** | **1.11** |  | **1.07** | **1.05** | **1.09** |  | **1.28** | **1.25** | **1.31** |  | **1.24** | **1.22** | **1.27** |  | **1.05** | **1.02** | **1.07** |  |  |
| Healthcare facilities | **1.15** | **1.13** | **1.17** |  | **1.12** | **1.10** | **1.14** |  | **1.08** | **1.06** | **1.10** |  | **1.29** | **1.27** | **1.32** |  | **1.24** | **1.21** | **1.26** |  | **1.02** | **1.00** | **1.05** |  |  |
| Physical activity facilities | **1.09** | **1.07** | **1.11** |  | **1.07** | **1.05** | **1.09** |  | **1.05** | **1.04** | **1.07** |  | **1.20** | **1.18** | **1.22** |  | **1.16** | **1.14** | **1.19** |  | **1.02** | **1.00** | **1.04** |  |  |
| OR: Odds ratio, CI: Confidence interval | | | | | | | | | | | | | | | | | | | | | | | |  |  |
| Neighborhoods are defined by buffer zones (radius 1000m) surrounding each person. Model 1 is unadjusted. Model 2 is adjusted for neighborhood-level deprivation. Model 3 is adjusted for neighborhood-level deprivation and individual-level age and income. | | | | | | | | | | | | | | | | | | | | | | | | | |
| Bold type: 95% CI does not include 1. | | | | | | | | | | | | | | | | | | | | | | | |  |  |
